# Beyond the Niche: Myelodysplastic Syndrome Topobiology in the Laboratory and in the Clinic

**DOI:** 10.3390/ijms17040553

**Published:** 2016-04-13

**Authors:** Eugenia Flores-Figueroa, Dita Gratzinger

**Affiliations:** 1 Oncology Research Unit, Oncology Hospital, National Medical Center, IMSS, Avenida Cuauhtémoc 330, Colonia Doctores, c.p. 06720 Mexico City, Mexico; 2 Department of Pathology, Stanford University School of Medicine 300 Pasteur Dr., L235, Stanford, CA 94305, USA

**Keywords:** myelodysplasia, myelodysplastic syndromes, topobiology, niche, mesenchymal stem cells, macrophages

## Abstract

We review the murine and human microenvironment and hematopoietic stem cell niche in the context of intact bone marrow architecture in man and mouse, both in normal and in myelodysplastic syndrome marrow. We propose that the complexity of the hematopoietic stem cell niche can usefully be approached in the context of its topobiology, and we provide a model that incorporates *in vitro* and *in vivo* models as well as *in situ* findings from intact human marrow to explain the changes seen in myelodysplastic syndrome patients. We highlight the clinical application of the study of the bone marrow microenvironment and its topobiology in myelodysplastic syndromes.

## 1. Introduction

Topobiology, first proposed in the setting of embryonic development [1], is the principle that cellular and molecular interactions are place-dependent. The bone marrow microenvironment is a complex topobiologic unit that orchestrates normal and, in the setting of myelodysplastic syndromes (MDS), malignant hematopoiesis. Perturbations of the microenvironment are capable of inducing myelodysplasia in mouse models, and translational studies indicate that evaluation of the microenvironment may provide novel prognostic information in myelodysplastic syndromes. The data that researchers acquire on this complex interdependent system are dependent on the limitations inherent in the assays. *In vitro* methods by their nature involve loss of microenvironmental interactions; mouse models have the advantage of a tractable and intact system, which does however differ in key ways from the human myelodysplastic marrow microenvironment. Herein we review bone marrow architecture in the context of the diagnostic bone marrow biopsy in man and in wild type mouse; the murine and human microenvironment and hematopoietic stem cell niche; and clinical applications of the study of the bone marrow microenvironment in myelodysplastic syndromes.

## 2. Bone Marrow Architecture in Man and Mouse

In the United States the median age of patients with MDS is 76, with more than 85% of patients with MDS aged over 60 at diagnosis [2]. The diagnosis and classification of myelodysplastic syndromes [3] is largely based on careful morphologic review of bone marrow biopsy and peripheral blood material for features of dysplasia and blast counts [4] in combination with cytogenetic studies. This diagnostic bone marrow biopsy typically comes from the posterior iliac crest, a flat bone that forms part of the pelvis. While in newborns the entire skeleton is marrow-forming, in adults over age 25 only the very proximal portion of tubular bones is hematopoietic, and the majority of hematopoiesis occurs in the axial skeleton [5]. Indeed a study of a population of older adults with a similar median age to that of MDS patients found the bulk of active marrow in the pelvis and vertebrae [6].

**Figure 1:**
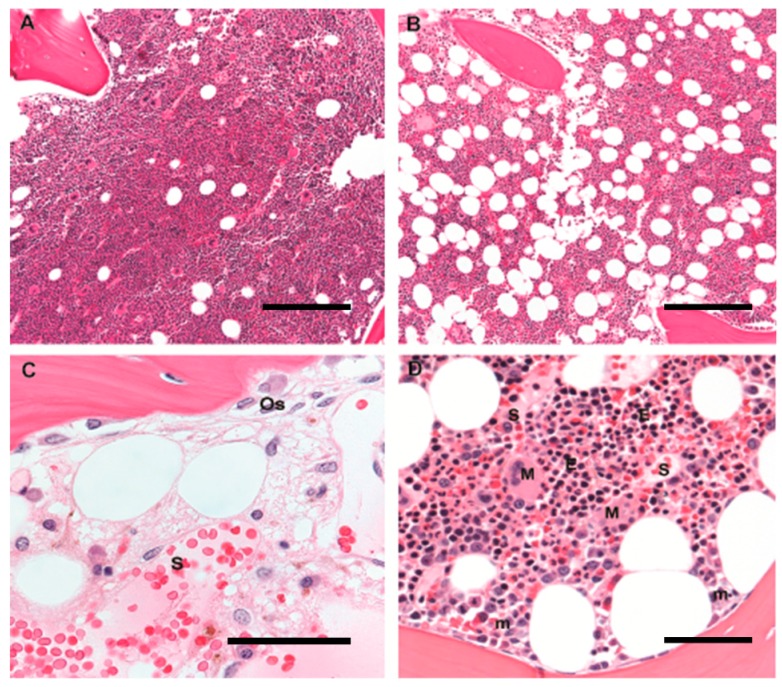
Human marrow architecture in youth and age: Hematopoiesis in human adults is predominantly axial, and diagnostic bone marrow biopsies sample the pelvic iliac crest. Trilineage hematopoiesis is admixed with increasing amounts of mature adipose tissue with age; adipocytes appear as round clear spaces. (**A**) A bone marrow biopsy from a 5-year old child is >90% cellular, with a predominance of trilineage hematopoiesis and little admixed adipose tissue; original magnification 10×; scale bar 100 μm; (**B**) A bone marrow biopsy from a 60-year old adult is composed of 50% hematopoietic elements and 50% admixed mature adipose tissue; original magnification 10×; scale bar 100 μm; (**C**) A post-chemotherapy-marrow reveals the underlying bone marrow architecture and microenvironment. Trabecular bone is curvilinear lamellar bone with apposed osteoblasts (Os), and a thin osteoid seam of unmineralized collagen. Dilated thin-walled sinusoids (S) are filled with red blood cells and have closely-apposed stromal cells with ovoid nuclei. Scattered mononuclear cells include plasma cells, mast cells, and macrophages, some of which contain yellow-brown hemosiderin pigment; original magnification 60×; scale bar 25 μm; (**D**) Erythroid colonies (E) appear as colonies of round cells with dark nuclei and are located away from trabecular bone, close to thin-walled sinusoidal vessels (S); megakaryocytes (M) are likewise located in close contact with sinusoids whereas immature myeloid precursors (m) are localized near trabecular bone. Original magnification 20×; scale bar 50 μm.

With aging comes a change in not just the distribution but the composition of the marrow: older adults gain marrow fat (“yellow marrow”) [7] resulting in a decrease from essentially 100% hematopoietic marrow in newborns to 40%–50% hematopoietic marrow, with 50%–60% marrow fat, in older adults in the age-range typically affected by MDS (Figure 1A,B). The subcortical marrow space is generally fatty-replaced in older adults, and in fact bony loss is associated with increased marrow adiposity [8]. Adult human iliac crest contains a relatively uniform inter-anastomosing network of trabecular bone—with a mean intertrabecular distance of 0.6–0.7 mm and mean trabecular plate thickness of 0.1 mm in older adults [9]. The specialized vasculature of bone marrow supports not just nutrient and oxygen delivery but also sites of egress for developing hematopoietic elements and specialized developmental niches. It consists of incoming arterioles with a coat of vascular smooth muscle and not infrequently associated plasma cells, capillaries with a diameter smaller than a red cell surrounded by pericytes, and a rich inter-anastomosing sinusoidal compartment consisting of a thin-walled and often gaping vascular network draining into the venous outflow. Erythroid colonies and megakaryocytes abut (closely neighbor) attenuated sinusoidal walls through which platelets and red blood cells are released into the circulation; immature myeloids abut trabecular bone and arterioles, and mature granulocytes exit into the sinusoids, sometimes by traversing megakaryocytic cytoplasm. (Figure 1C,D) [10,11,12].

**Figure 2:**
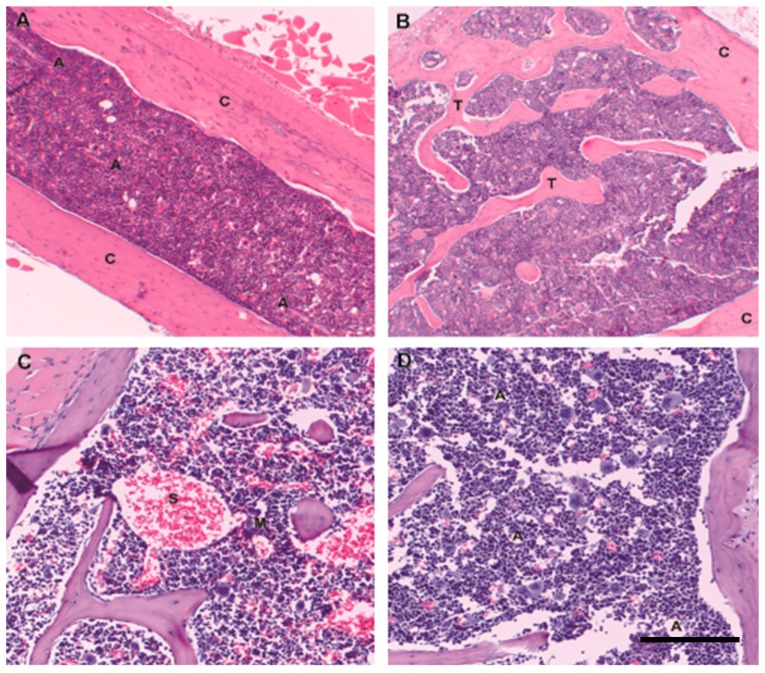
Mouse marrow architecture in youth and age: Hematopoiesis in mice is present throughout the skeleton. Photomicrographs from C57BL6 wild-type animals. (**A**) Mouse hematopoietic marrow architecture is most commonly evaluated in whole mounts of tubular bone (femur), where predominantly cortical bone (C) in contact with hematopoietic marrow. Small-diameter arterioles (A) with well-defined multilayered walls run the length of the femur; (**B**) Flat bone sites such as pelvis in contrast more closely resemble the trabecular-bone (T) predominant architecture of adult human hematopoietic marrow; cortical bone, (C); (**C**) Mouse sternum also has a significant trabecular component; in 8 week-old mice hematopoietic cellularity is close to 100%, with no identifiable admixed fat. Similar to human marrow megakaryocytes (M) are closely opposed to thin-walled and sometimes ectatic sinusoidal vessels (S); (**D**) Similar to human marrow, mouse marrow increases in fat content with age, although the absolute percentage of mature adipose tissue is much lower than that seen in older adult humans. Here a two-year old mouse shows scattered admixed adipocytes (A), accounting for 5%–10% of marrow cellularity. Original magnification 4×; scale bar 200 μm.

In contrast to the human diagnostic bone marrow biopsy, that of the mouse is generally a whole mount of a tubular bone (femur) in a relatively young animal (Figure 2A). In contrast to older adult human hematopoietic marrow the proportion of marrow fat is generally lower, and the predominant form of bone in contact with hematopoietic marrow is cortical rather than trabecular. Cross-sectional mid-femoral marrow area varies from 0.5–1 mm^2^ depending on strain [13]; this translates to a diameter of 0.8–1.1 mm, about 1.5 to 2 times the typical intertrabecular distance in adult human iliac crest. In mouse this intercortical marrow area is bisected by the central nutrient artery and vein running the length of the femur, such that all areas of the hematopoietic marrow are maximally 0.4–0.6 mm from the nutrient artery and vein, a topobiologic feature lacking in humans and that may have species-specific consequences in terms of nutrient density and oxygenation status of marrow microdomains. Murine flat bones such as the pelvis (Figure 2B) and sternum (Figure 2C,D) more faithfully recapitulate the trabecular bone-predominant architecture of human axial bone. Positioning of erythroid colonies, myeloids, and megakaryocytes with respect to the marrow vasculature is similar in mice and humans, and the distribution of maturing myeloid and erythroid precursors is within the range seen in human marrow [14]. Marrow adiposity and bone volume vary across mouse models, and in mouse as in human marrow adiposity increases with age [15], although absolute mature adipose tissue content in aged mice remains lower than that in older adult humans (Figure 2C,D).

## 3. The Niche/Microenvironment in Drosophila and in the Mouse

### 3.1. The Stem Cell Niche Concept

The niche concept was adapted from the field of ecology to the stem cell field by Ray Schofield in 1978 [16]. The naturalist Joseph Grinell was the first author to use the term “niche” in 1917 to describe the sum of the habitat requirements that enables a species to persist and reproduce [17]. The term was popularized by other zoologists [18], who stressed the physical as well as the biological requirements of the species. They recognized the reciprocal influence of the organism on the niche and the niche on the organism.

Experiments in mouse showed that the potential of hematopoietic stem cells (HSCs), evaluated as their ability to form colonies in the spleen of irradiated animals, was gradually lost after serial transplantation. These findings led Ray Schofield to postulate that HSCs must remain in the bone marrow in a specific site (niche) strongly associated to “other cell types” which regulate their physiology and protect them from inductive (maturation or proliferation) signals [16]. Although the concept for a stem cell niche arose from the mouse hematopoietic system, the complexity of the HSC niche is such that the first niche to be described was in fact not hematopoietic or even mammalian.

### 3.2. Drosophila Germinal Stem Cell Niche, a Fixed Niche

The first niche to be described, and also one of the best characterized, is the Drosophila female gonadal stem cell niche (fGSC-niche) [19]. We will briefly describe this prototypical and topobiologically simple niche in order to introduce concepts applicable to the HSC niche. The fGSC-niche is highly tractable due to (a) its fixed location in the germarium at the tip of the female ovariola; (b) the fixed position of the gonadal stem cells (GSCs); (c) the small number of niche cells and (d) the capacity to morphologically track and individually target both stem and niche cells [17]. The niche cap cells are in direct contact with the stem cells through adherens junctions mediated by E-cadherin and produce several morphogens. Cap cells are essential for the maintenance of stem cells and determine their fate in a spatially distinct manner. After a stem cell divides, daughter cells that retain contact with cap cells self-renew, whereas daughter cells that lose contact with cap cells differentiate into cytoblasts. Terminal filament and cap cells niche cells secrete factors (Piwi/Yb and Hedgehog) that promote stem cell self-renewal. Terminal filament cells also control niche size indirectly through Notch ligand Delta, which induces neighboring somatic cells to differentiate into cap cells [17]. The Drosophila germinarium system is a prototypical developmental niche that: resides within a fixed structure; has a small fixed number of niche cells and a small number of stem cells; and shares molecular pathways (cadherins, Notch, Hedgehog) with other niches including the HSC niche.

### 3.3. The Hematopoietic Stem Cell Niche in the Mouse, a Dynamic and Complex Niche

It has been almost forty years since Schofield first postulated the existence of the HSC niche. Since the turn of this century, knowledge in this field has begun growing exponentially. The study of the HSC niche had to overcome technological challenges, including the lack of specific markers for both HSC and niche cells, the low frequency of HSCs, and the development of microscopy methods to visualize live cells inside the bones. Before 2004, the field lacked specific HSC markers for their *in situ* identification, so the evidence of an HSC niche was indirect [20,21]. Studies from Scadden and Li’s laboratories concluded that HSCs were located at endosteal niches, as the manipulation of osteoblasts had a direct impact on HSC number and differentiation potential [20,21,22,23]. However, the development of new HSC markers and their direct visualization [24] by Dr. Sean Morrison’s group allowed the specific localization of HSCs to perivascular areas, (approximately 85% of HSCs reside within 10 μm of a sinusoidal blood vessel) and at a lower frequency in the endosteal areas in mice.

### 3.4. HSC Niche Is Dynamic

In contrast to the fixed fGSC-niche in *Drosophila* the HSC niche is dynamic; it changes over the course of development and in response to various stimuli. After birth, the HSC niche is located primarily in the bone marrow, a tissue that is constantly remodeling to fulfill calcium requirements and repair microscopic lesions. An adult skeleton is completely replaced every ten years, which means that 10% of the skeleton is being remodeled at any given time [25]. Bone remodeling has an impact on many cell types, not just osteoblasts and osteoclasts. Teufel *et al.* [26] showed that pharmacologic inhibition of bone remodeling in the mouse led to a decrease not only in the number of osteoblasts but also plasma cells and megakaryocytes.

The presence of HSCs in both perivascular and, to a lesser extent, endosteal niches may indicate a dynamic transition of HSCs within the bone marrow and/or reflect a different cell cycle status or different reconstitution potential. Kunisaki Y *et al.* has described that HSCs located closer to arteries are quiescent [27]. Supporting this hypothesis, Guezguez *et al.* [28] reported that HSC reconstitution potential varies according to location within the bone (trabecular *vs.* long bone area). HSCs residing at the bone epiphysis showed greater long-term reconstitution potential. In contrast, Dr. Morrison’s group showed that both proliferating and non-proliferating HSCs reside in the sinusoidal perivascular area but not adjacent to arteries. The use of different markers and models may underlie apparent discrepancies among the studies; this highlights the need for standardized markers and models and as well as a better understanding of developmental and species and strain-specific variation.

### 3.5. HSC Niche Is Complex

The fGSC niche in Drosophila has just three niche cell types; in contrast, there are multiple cells that regulate murine HSC physiology; osteoblasts [20,21], endothelial cells [29,30,31], mesenchymal stromal cells [29,32,33], macrophages [34,35,36], cells of the nervous system [37], megakaryocytes [38,39], osteoclasts [40,41], and lymphocytes and other hematopoietic elements [42]. HSCs do not have a direct contact to all this cell types, but the studies have demonstrated an indirect participation. Plasma cells are also perivascular/associated with mesenchymal stem cells (MSCs), and it has been reported that they share the niche with HSCs [43]. Although it has not yet been reported that plasma cells contribute to the niche, they are well positioned to do so.

MSCs are one of the niche cells that have direct contact to HSCs [24], and have gained a lot of attention in recent years. In 2010 two papers using *in vivo* models demonstrated their key participation in the niche [32,33]. The conditional depletion of Nestin+ MSCs causes a deficiency in the recruitment of HSCs to bone marrow [33], and the depletion of CXCL12+ (C-X-C motif chemokine ligand 12) MSCs decreases the number of both HSCs and B-lymphoid progenitors [32]. It has to be taken into consideration that both models depleted other cell types that express nestin or CXCL12, including endothelial cells and osteoblasts, respectively.

To date there are several markers that have been used to track and target MSCs in mice, including nestin [33], CXCL12 [32], PRx-1 (Periredoxin 1) [44], and Leptin receptor (LepR) [45], among others. The complexity of the niche is reflected by the number of molecules that regulate HSC physiology, including cytokines [46], chemokines [32], morphogens [31], adhesion molecules [21], and extracellular matrix proteins [47]. Those molecules are each produced by several cell types, so in order to assess which are key cell types in the niche, several authors have been conditionally depleting production of these factors by a single cell type. Greenbaum and colleagues (from Nagasawa’s group) undertook the task of specifically eliminating subpopulations of cells expressing CXCL12: osteoblasts and osteoprogenitors, endothelial cells and cells expressing MSC marker Prx-1 [44]. They found that CXCL12 expression by endothelial cells and MSCs influences the development and localization of HSCs. Dr. Morrison’s group used a model of differential depletion of stem cell factor (SCF, also known as KIT-ligand) in osteoblasts, hematopoietic cells, endothelial cells and LepR+ MSCs; these authors found that HSC were eliminated in the bone marrow of mice whose SCF had been eliminated in endothelial cells and MSCs only [29].

### 3.6. The HSC Niche Is Not Exclusive

Key players in the hematopoietic stem cell niche also play other roles in the marrow. MSCs for example may contact not only HSCs but also progenitor and maturing myeloid cells as well as other cell types (plasma cells, lymphoid progenitors, megakaryocytes, adipocytes, vasculature, *etc.*). This model differs from the fGSC in Drosophila where the cap cells do not contact mature cells. It has been demonstrated that B cell progenitors (hematogones) are located along MSCs [32] according to their differentiation status, which has also been demonstrated for humans [48]. MSCs are elongate and have cytoplasmic extensions that contact multiple cells and cell types simultaneously, including HSC and other niche cells.

## 4. The Niche/Microenvironment in Humans

As in mouse, the bone marrow microenvironment in human is complex and consists of bone and its lining cells, osteoblasts, and osteoclasts; vasculature, including vascular smooth muscle-clad arterioles, pericyte-clad capillaries, and thin-walled sinusoids; mature adipose tissue; MSCs; fibroblasts; nerve fibers; macrophages; and resident mature cell types such as plasma cells, mast cells, and lymphocytes; hematopoietic elements themselves, including megakaryocytes, maturing myeloids, and erythroid colonies also contribute to the microenvironment. Any individual cell within this rich three-dimensional environment may make direct contact with multiple cell types and receive biochemical information from more through surface-displayed, secreted or otherwise transferred bioactive compounds. Many of these cell types have been shown to play important roles in hematopoietic stem cell biology in the mouse model, as discussed above. Much less is known about their distribution with respect to hematopoietic stem/progenitor cells, and of the distribution and contact of microenvironmental components with each other, in intact human marrow.

Some important microenvironmental components such as osteoblasts, mature adipose tissue, arterioles and megakaryocytes are readily identifiable morphologically in intact bone marrow core biopsies. Others—especially those with a low nuclear:cytoplasmic ratio and attenuated cytoplasmic processes, such as MSCs, pericytes, nerve fibers, macrophages, and collapsed sinusoidal vessels—are not easily identifiable by light microscopy alone. Immunohistochemistry and immunofluorescence can be used to delineate the MSC compartment, vasculature, and macrophages in intact human bone marrow and to document the relationships among them. Low affinity nerve growth factor receptor, or CD271, was described over 20 years ago as identifying a delicate ramifying stromal cell population in human marrow [49] and has more recently been widely used to identify and isolate multipotent human mesenchymal stem cells [50]. Immunoelectron microscopy studies [51] and later immunofluorescence studies [52] noted proximity of hematopoietic progenitor/stem cells (HPSCs) to delicately ramifying CD271+ MSCs in benign human marrow.

We analyzed the distribution of CD271+ MSCs in intact benign, MDS, and AML bone marrow core biopsies [53]; in both benign and malignant marrow we noted that extensively ramifying CD271+ MSCs were present throughout, and were enriched in a perivascular and paratrabecular location. Each individual MSC has numerous cell-cell contacts through its long slender cytoplasmic extensions; each individual MSC may have one, two, or occasionally three of these extensions visible within a single plane of section. While in a single two-dimensional plane of section we noted only occasional MSC-MSC contacts along the axon-like cytoplasmic extensions, it is clearly possible that within the quite large three-dimensional effective volume of these arborizing cells there are numerous MSC-MSC contacts creating an MSC network throughout the hematopoietic marrow. Those MSCs closely apposed to bone marrow vasculature or trabecular bone have long extensions that parallel these structures; the relationship to the vascular endothelium differs by vessel type, as sinusoidal endothelium is in direct contact with MSCs whereas capillaries and arterioles are separated from their MSC layer by tightly apposed pericytes/vascular smooth muscle. These vascular compartments are functionally distinct; in mice Sca1 and VEGFR3 have been reported to selectively identify the arteriolar and sinusoidal compartment, respectively [54]. In the intact human bone marrow we have found that nestin selectively labels capillary and arteriolar endothelium, and CD105 (endoglin) selectively labels sinusoidal endothelium [53,55].

**Figure 3:**
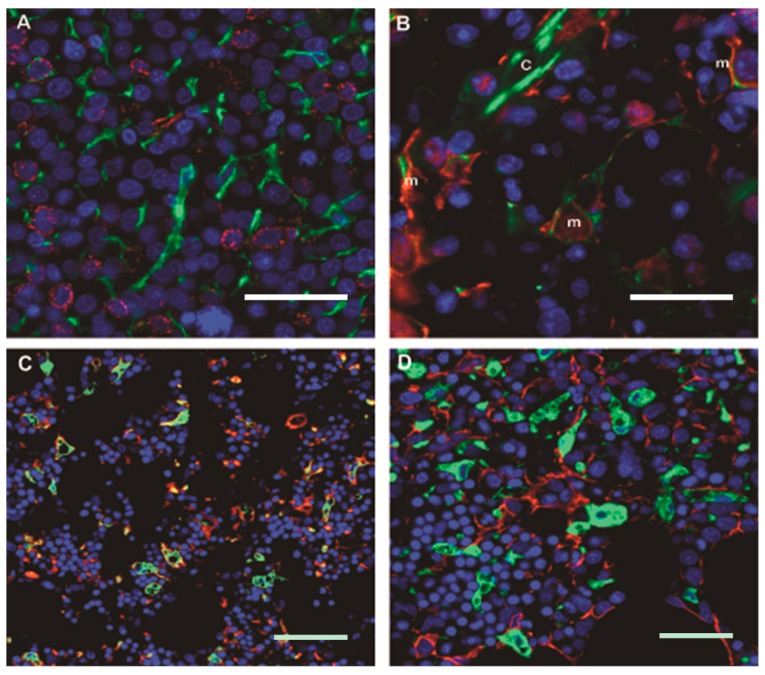
Human bone marrow microenvironment: CD271+ mesenchymal stromal cells form a delicate arborizing reticular network that is enriched around vasculature, are in contact with the majority of CD34+ hematopoietic progenitor/stem cells, and coexpress the chemokine CXCL12. Bone marrow iron is stored within HO1-expressing CD163+ macrophages, which are also in contact with CD271+ MSCs. (**A**) Double immunofluorescence (DIF) for CD271 (green) and CD34 (red) with DAPI-counterstained nuclei (blue) in bone marrow involved by MDS, refractory anemia with excess blasts-2. Note the close apposition of delicate MSC cytoplasmic extensions with CD34+ blasts; original magnification 60×; scale bar 25 μm; (**B**) DIF for CD271 (red), CXCL12 (green), nuclei (blue) in bone marrow involved by MDS, refractory anemia with isolated deletion 5q. Note CD271+ MSCs (m) that are positive for CXCL12, as well as a brightly CXCL12+ capillary (c) with closely apposed CD271+ MSCs; original magnification 60×; scale bar 25 μm (**C**) DIF for heme oxygenase-1 (green), CD271 (red) and nuclei (blue) in bone marrow involved by MDS, refractory anemia. Within the intact marrow, heme oxygenase-1 (HO1) is largely restricted to CD163+ macrophages, which contact numerous cells around their main body and through stellate projections; original magnification 20×; scale bar 50 μm; (**D**) DIF for HO1 (green), CD271 (red), and nuclei (blue) in bone marrow involved by MDS, refractory anemia with excess blasts-1. Note the intimate proximity of HO1+ macrophages and CD271+ MSCs original magnification 40×; scale bar 25 μm.

Where within this vascular and MSC-rich microenvironment might we find the so-called “niche” for hematopoietic precursors? We found that CD34+ HPSCs tended to be located within a few cell diameters of marrow vasculature, with close to half of HPSCs within 10 μm of vasculature in both benign and MDS marrow. Double immunofluorescence reveals that CD34+ HPSCs are in direct contact with CD271+ MSCs (Figure 3A); the slender processes of the MSCs contact and in some instances can be seen to follow the contour of the HPSC in an intimate embrace. We found that in benign and MDS marrow alike the vast majority (>80%) of CD34+ HPSCs were in direct contact with CD271+ MSCs in a single two-dimensional plane of section; it is likely that if the full volume of the HPSCs were examined, all would be found to be in direct contact. As discussed above, in mouse models, CXCL12-abundant reticular cells, or CAR cells, are crucial components of the HPSC niche [32] and *in vitro* studies on HPSCs confirm the importance of signaling via the CXCL12 receptor CXCR4 in the human setting [56]. Indeed, we found that MSCs in intact human marrow variably express CXCL12 (Figure 3B); strong expression is also seen in endothelial cells. While CD34+ HPSCs are in direct contact with CXCL12-expressing MSCs, they are separated from endothelium by densely-packed pericytes/vascular smooth muscle at the level of capillaries. At the level of sinusoids however CD34+ HPSCs may encounter the endothelial layer directly or via a thin closely-apposed layer of MSC cytoplasm.

Any study of the bone marrow microenvironment must take into account the ongoing iron handling that is a requirement for successful erythropoiesis but that also exposes marrow cells to the risk of oxidative damage. The breakdown of heme is accomplished within bone marrow macrophages, where heme oxygenase-1 (HO1) acts to safely free iron which can then be stored inertly within ferritin aggregates known as hemosiderin [57,58]. In mouse models bone marrow macrophages promote retention of HPSCs [59] and HO1 is involved in the control of myeloid maturation [60] and the differentiation potential of MSCs [61]. Through quantitative image analysis of intact human bone marrow we have determined that the vast majority (>99%) of marrow HO1 is in fact contained within CD163+ marrow macrophages (Figure 3C), where it colocalizes with marrow iron and ferritin [62]. Interestingly, HO1+ macrophages are in frequent and extensive contact with CD271+ MSCs (Figure 3D), providing a locus for potential HO1-mediated effects on CD271+ MSCs and, via MSCs, on associated CD34+ HPSCs. In fact, such intimate MSC-macrophage interactions have been recently demonstrated *in vitro*, where MSCs were shown to control nearby macrophages through exosome-mediated transfer of microRNAs and to control MSC oxidative stress by off-loading damaging mitochondria to macrophages [63].

Mouse studies provide an amazing resource for understanding of the human bone marrow microenvironment; nevertheless interspecies differences must be anticipated both in topobiology and phenotypes. In addition to differences in relative composition of the microenvironment—such as admixed mature adipose tissue, and trabecular bone-predominant architecture—we must beware immunophenotypic differences between species. For example, nestin is an intermediate filament protein that in mouse models identifies a mesenchymal stem cell population that forms a functional component of the hematopoietic stem cell niche [33]. In intact adult human marrow however the predominant nestin+ population is capillary and arteriolar endothelium [53,55]. CD105 (endoglin) in contrast identifies the human sinusoidal endothelial compartment in the intact marrow [55].

CD146 (melanoma cell adhesion molecule) has been implicated in reconstitution of the hematopoietic stem cell niche: CD146+ human mesenchymal cells were shown to be capable of reconstituting and transplanting the hematopoietic microenvironment in xenotransplanted mice [64]. The counterpart of these cells in the intact marrow of the older adults who represent the majority of those at risk for MDS is unclear. In the intact marrow of older adults CD146 is most brightly expressed in the vascular smooth muscle/pericytes of capillaries and arterioles, with dimmer endothelial expression and variable expression in adipocytes [53]. Indeed, the proportion of CD271+ mesenchymal stem cells expressing CD146 drops of significantly with donor age [65]. Tormin *et al.* have demonstrated that both CD146+ and CD146− CD271+ mesenchymal stem cells can reconstitute the hematopoietic microenvironment, and that CD146 is downregulated under conditions of hypoxia [52]. Laboratory models of the hematopoietic microenvironment are likely to use relatively young mice and potentially young human mesenchymal stem cell donors, and also, given the small size and different vascularity of the model system, may not replicate the oxygen tension present in the flat bones that contain most of the hematopoietic marrow of the adult human.

Murine studies implicate neural signaling [66] in hematopoietic stem cell release [37] and quiescence [67]; marrow innervation in mice is part of the arteriolar neurovascular bundle, where innervation is primarily associated with the vascular smooth muscle and adventitium [68]. The topology is different in adult human flat bone, where by definition no longitudinal neurovascular bundle exists, and there is proportionately little hematopoietic marrow contact with cortical bone at the site of entry of neurovascular structures. Little is known about the innervation pattern of human marrow; very rare individual neurofilament+ nerve twigs are present in association with scattered arterioles (personal observation, DG). Whether these represent a site of quiescent hematopoietic stem cell maintenance in humans, during development or throughout life, is unclear, and points to the need for rigorous studies of human marrow microarchitecture. Lacking as well is a simple way to identify and differentiate human hematopoietic stem cells *in situ*, much less functional subsets of hematopoietic stem cells.

Also remaining to be clarified *in situ* is the reciprocal relationship of hematopoietic progenitor cells to B cell precursors, or hematogones. In intact human marrow hematogones are arranged in a spatially oriented manner along CD10+ stromal cells with maturation toward sinusoids [48]. Interestingly, hematogones are decreased in the marrow of patients with MDS [69,70], even as dysfunctional hematopoiesis fills the marrow and hematopoietic progenitors become more frequent. Work in mice implies that MSCs and other microenvironmental cells can be reprogrammed from a B-lymphoid-supportive phenotype to a myeloid-supportive phenotype in response to growth factor input [71]. The two types of progenitors may compete for limited numbers of permissive niches; alternately, given the expansion of the MSC compartment in MDS, a global change in the microenvironment due to reciprocal dysfunctional interactions among MDS HPSCs, MSCs, macrophages, and maturing hematopoietic elements may conspire to decrease support of lymphopoiesis throughout the MDS marrow.

## 5. The Niche/Microenvironment in MDS

Initial studies of the hematopoietic microenvironment in MDS patients were based on the *in vitro* culture of stromal cells using the long-term marrow culture (LTMC) system. Most studies did not find any abnormalities in either proliferation of MSCs or their potential as measured by colony forming unit-fibroblast (CFU-F) frequency [72,73,74,75,76]. In contrast, several studies have reported functional abnormalities in the stroma of MDS patients [77,78], including increased secretion of pro-inflammatory cytokines (tumor necrosis factor alpha/TNFα, interleukin 1 beta/IL-1β, interleukin 6/IL-6, interferon gamma/IFNγ and transforming growth factor beta/TGFβ) [73,75,79,80,81,82]. Regarding the hematopoietic support capability of MDS–derived stromal cells *in vitro*, the results are contradictory, as both normal [76] and deficient [77] hematopoietic support has been reported.

Altered cytokine expression by MDS marrow-derived macrophages has more uniform support in the literature, including TNFα [75,80,83] and, in a subset of patients, IFNγ [80]. The number of monocyte/macrophages has also been reported to correlate with increased levels of TGFβ in MDS bone marrow biopsies [84]. Conditioned media from MDS-derived macrophages inhibited the growth of myeloid progenitors (colony forming unit-granulocyte macrophage/CFU-GM) [83]. MDS patients also have aberrant iron handling and a propensity to iron overload that may alter macrophage function. Nybbaken G *et al.* [62] found that macrophage iron is increased in MDS independent of transfusion status; CD163+ macrophage density, heme oxygenase-1 (HO-1) and H-ferritin expression increased in tandem with marrow iron. High macrophage HO-1 was in turn associated with shorter survival independent of International Prognostic Scoring System-Revised (IPSSR) [85] and transfusion history in MDS patients.

### 5.1. Mesenchymal Stromal Cells in MDS

There has been a lot of attention paid to the role of MSCs in normal and dysplastic/leukemic hematopoiesis, in both mouse and human [86,87,88,89], The first papers that characterized MSCs in the setting of MDS by morphology, immunophenotype and differentiation potential were published in 2005 [90] and since, more than 80 papers have been published in this field (Table 1).The first finding was related to the chromosomal abnormalities present in MSCs. In this regard, Flores-Figueroa *et al.*, first described that MSCs from MDS patients harbor chromosomal abnormalities in 55.5% of the patients [90]; Blau *et al.* corroborated this finding in 44% of their cohort [91]. As both studies analyzed cultured MSCs, it was not clear if the chromosomal abnormalities were preset before or were gained after *in vitro* culture. Lopez-Villar *et al.* analyzed the genomic changes of uncultured MSCs (CD271+CD73+CD45−CD34−) by array comparative genomic hybridization (CGH) studies, and found that the alterations were indeed present before the cells were cultured [92]. It is noteworthy that in all three studies, the patients who harbor chromosomal/genomic alterations in the MSCs also have distinct cytogenetic alterations in their hematopoietic cells.

All of the cytogenetic studies of MSCs, including a pioneer study by Soenen-Cornu [93], found that HSCs and MSCs do not harbor the same cytogenetic abnormalities. There are still many questions that need to be elucidated: the etiology of the chromosomal anomaly in MDS-MSCs; whether there exists a common progenitor of hematopoietic and mesenchymal stromal cells; whether some particular agent causes genomic instability in both hematopoietic cells and in MSCs; whether genetically unstable HSCs induce genetic instability in MSCs, or *vice versa*; or alternately whether these patients harbor germ line mutations in genome stability genes. Sequencing the genome of both hematopoietic stem cells and MSCs from the same patients could help elucidate the pathophysiology underlying this relationship between unrelated cytogenetic lesions in MSCs and HSCs.

Most subsequent studies regarding MDS-derived MSCs have focused on their functional properties, including their frequency, proliferation, differentiation, hematopoietic support and immunomodulatory functions. Most of the studies (Table 1) have painted a picture of decreased potency in MDS-derived MSCs, including decreased CFU-F and proliferation potential together with increased senescence of MSCs [92,94,95]. This is in marked contrast to *in situ* studies that have measured the number of MSCs in bone marrow biopsies from MDS patients [53,96,97], all of which have instead reported an increased density and number of MSC (CD271+). These contradictory results may be an artifact of culture conditions, which cannot substitute for the complex microenvironment of intact bone marrow. Alternately *in vitro* experiments may identify and characterize a functional subset of MSCs that has not yet been distinguished morphologically and immunophenotypically *in situ*. Discrepancies between *in vitro* studies and studies of intact human tissue aside, *in vitro* studies of MSCs themselves vary markedly with respect to their culture conditions (number of passages, media), the heterogeneity of the MDS subtypes studied, and the nature of the control samples (which ideally should be age-matched and not involved by other pathology). Moreover, MSCs are a highly heterogeneous population, with for example variable CD146 and CD10 expression. These subpopulations may have different functional properties, which may be represented in different percentages from study to study.

MSCs are defined by their ability to differentiate into bone, fat, and cartilage *in vitro*. Most studies agree that MDS-derived MSCs have defective osteogenic potential [94,95,98,99,100,101] analyzed in *in vitro* cultures. These results are in agreement with a histomorphometric study performed by Mellibovsky *et al*. [102]. The authors reported decreased osteoblasts and osteoclasts number in bone marrow biopsies from MDS patients. Regarding adipogenic potential however, there is no consensus; almost half of the studies report normal adipogenic potential [90,103,104,105] and the rest, either higher [106] or decreased [94,99]. The number of adipocytes is generally decreased in the bone marrow of MDS patients except in the relatively rare hypocellular MDS. In general this is due to expansion of the ineffective hematopoietic compartment, which is generally dominated by increased/expanded erythropoietic colonies. It is unclear therefore whether there is a true decrease in the ability of MSCs to differentiate toward adipocytes, or simply competition for marrow space by the hyperproliferative hematopoietic compartment.

The immunoregulatory mechanisms of MSCs has been thoroughly investigated because of their impact on the pathophysiology of many diseases and because of their potential use in cellular therapy [107]. Literature has reported that MDS-MSCs have increased immunomodulatory properties, including potent inhibition of dendritic cell differentiation and function [108], and increased production of immune-dampening regulatory T cells (Tregs) [109]. Together these microenvironmental immunosuppressors appear to protect the floridly aberrant MDS hematopoietic compartment from immune surveillance and elimination.

### 5.2. Mouse Models to Study the Role of the Hematopoietic Niche/Bone Marrow Microenvironment in Hematopoiesis and MDS

The Steel (S*l*/S*l*^d^) and white-spotting (W/W^v^) mutated mice were the first *in vivo* models to highlight the key role of the microenvironment in hematopoiesis. Both mice shared the same phenotype of macrocytic anemia, but they were cured by different approaches. Steel mice were cured by surgical spleen implants from a wildtype mouse, whereas white-spotting mice were cured by intravenous injection of wild type marrow cells. Dr. Michael Dexter found that adherent microenvironmental cells from steel mice were able to support the normal differentiation of steel mice marrow cells [110,111], supporting the idea that abnormalities in both the hematopoietic compartment and their microenvironment may lead to abnormal hematologic conditions. We now know that steel mouse is characterized by mutations in the stem cell factor (SCF), which is produced by stromal cells, and white-spotting mice by mutations in c-kit, a receptor present in HPSCs.

In spite of the evidence of the key role of the microenvironment in hematopoiesis, strong evidence for their role in abnormal hematopoiesis was still lacking until two seminal papers published in Cell by Louise Purton and Stuart Orkin’s groups in 2007 [112,113]. Both groups found that alterations present solely in the microenvironment led to myeloproliferation. Both groups used chimeric transplantation of wildtype bone marrow cells into mutated recipients (retinoic acid receptor gamma/RARγ and retinoblastoma/Rb mutations, respectively) and mutated cells (RARγ or Rb) into wild type hosts to elucidate that the phenotype was caused by alterations in the microenvironment. 

**Figure 4:**
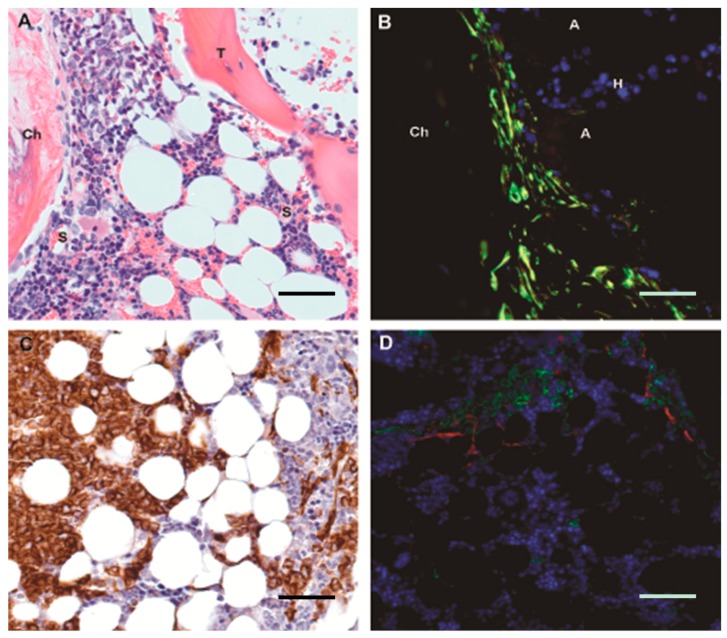
Recapitulating the human bone marrow microenvironment in mouse: A subcutaneous ossicle with mesenchymal components derived from human mesenchymal stromal cells is seeded by murine trilineage hematopoiesis and provides an easily accessible niche for xenotransplanted human myeloid neoplasms. (**A**) The overall ossicle architecture is similar to adult human bone marrow architecture, including trilineage hematopoiesis admixed with significant mature adipose tissue, interspersed trabecular bone (T), and visible sinusoids (S); the chondroid cap (Ch) which is the residuum of the initial chondrogenic MSC outgrowth is visible at the end of the ossicle; original magnification 40×; scale bar 25 μm; (**B**) Double immunofluorescence (DIF) for species non-specific vimentin (green), human-specific vimentin (red) and nuclei (blue) in a subcutaneous ossicle. The partially calcified chondroid cap (Ch) abuts admixed murine hematopoiesis visible as blue nuclei (H) and mature adipose tissue (A). All mesenchymal-derived cells, including chondrocytes and bipolar cells, are human vimentin-positive as well as species-nonspecific vimentin-positive, resulting in a yellow-green color; no red mouse-derived mesenchymal population is present, confirming that the microenvironment is entirely human-derived; original magnification 40×; scale bar 25 μm; (**C**) Immunohistochemistry for human vimentin in an ossicle seeded with human acute myeloid leukemia. Human vimentin-positive arborizing stromal cells are visible within residual murine hematopoiesis, as are numerous human-vimentin positive leukemic blasts; original magnification 40×; scale bar 25 μm; (**D**) DIF for species human CD45 (green), human CD271 (red) and nuclei (blue) in a subcutaneous ossicle with xenotransplanted acute myeloid leukemia. Recapitulating the situation in human bone marrow, human myeloid blasts are associated with human-derived CD271+ MSCs original magnification 20×; scale bar 50 μm.

The strategy used in these papers was a turning point for the field and paved the way for the developing concept of a tumorigenic or cancer niche/microenvironment [114]. In MDS evidence for the role of the microenvironment/niche in the pathophysiology of the disease has been strengthened by murine models that demonstrated that alterations in the splicing mechanisms of MSC lead not only to myeloproliferation, but to dysplasia and malignant transformation [115]. Dr. Vivienne Rebel has recently published an excellent review of mouse models of MDS [116]. We will briefly highlight xenotransplant mouse models that recapitulate the abnormalities seen in the MSCs of MDS patients. MSCs have also been shown to be necessary for the engraftment of MSCs in xenotransplant models [117,118]. Transplantation of human MSCs into immunodeficient mice can support recapitulation of hematopoietic marrow microenvironment containing human-derived vasculature, bone, and other mesenchymal cell types [119]; only bone-marrow-derived MSCs are capable of recapitulating the human hematopoietic niche and supporting both murine and human hematopoiesis [120]. Subcutaneous xenotransplantation of human MSCs supports formation of an accessible “ossicle” with improved ability to transplant human myeloid neoplasms [121]. Overall low power marrow architecture in the ossicle is quite similar to that in adult human flat bone, including presence of trabecular bone, trilineage hematopoiesis with significant admixed mature adipose tissue, and visible sinusoidal vasculature. The mesenchymal components are entirely human-derived and include CD271+ MSCs which associate with transplanted human myeloid blasts (Figure 4), recapitulating the situation in intact human marrow.

## 6. Clinical Application of the Study of Niche/Microenvironment

Myelodysplastic syndromes are quite variable in their hematologic manifestations, cytogenetics, and clinical course. Currently classification of MDS is based on several features of the hematopoietic compartment—a combination of morphologic dysplasia, blast percentage in the peripheral blood and bone marrow, presence or absence of aberrant iron accumulation within erythroid precursor mitochondria (ring sideroblasts), and cytogenetic abnormalities [3,122]. Subsequent classifications are likely to incorporate MDS-related molecular abnormalities [123] although the presence of these mutations must be interpreted in the setting of the complete clinicopathologic picture as such mutations are not specific to MDS or even to hematologic malignancy.

The prevalence of detectable clonal hematopoiesis with the same somatic mutations seen in MDS, such as *DNMT3A*, *ASXL1*, and *TET2*, is quite high in otherwise healthy older adults—one study demonstrated a prevalence of 10% in patients older than 65, a very small proportion of whom go on to develop hematopoietic malignancy [124]. Nevertheless as our experience with next generation sequencing-based molecular studies of the hematopoietic compartment matures we can anticipate that the definition of MDS may broaden to include a subset of patients with so called idiopathic cytopenia of uncertain significance (ICUS) [125] based on demonstration of certain combinations of molecular lesions in the setting of chronic otherwise unexplained cytopenias [125]. Clearly however the presence of clonal hematopoiesis, even with acquired mutations that are enriched in myeloid neoplasia, is insufficient on its own to support full-blown manifestation as MDS. Compared to molecular study of the human hematopoietic compartment, which can be readily sampled in large cohorts and tracked over time in individual patients based on peripheral blood specimens, study of human bone marrow mesenchymal stromal cells and other microenvironmental components is in its infancy.

Based on mouse studies we would anticipate that perturbation of the MSC compartment may contribute to the development of MDS in older adults who are at increased risk due to existing clonal hematopoiesis. Tantalizingly, some of the same epigenetic regulators that are recurrently mutated in hematopoietic cells in clonal hematopoiesis and myeloid neoplasia appear to be involved in controlling proliferation, pluripotency, and differentiation potential in mesenchymal stem cells. For example, TET1/2 epigenetically regulates expression of insulin-like growth factor 2 mRNA-binding protein 1 (IGF2BP1) [126], an oncogene with multiple regulatory targets including *c-MYC.* The DNA methyltransferase DNMT3A regulates mesenchymal stem cell differentiation toward endothelial cells [127], with potential effects on the perivascular hematopoietic stem cell niche. 

Recently mouse studies have shown that restoration of a wild type bone marrow microenvironment slows progression of MDS to acute leukemia [128]; it is to be hoped that targeting of the marrow microenvironment may similarly slow the progression of human disease. In fact, we may already be taking advantage of this effect inadvertently: DNMT3A, which was discussed above as a regulator of mesenchymal stem cell differentiation, is a target of the front-line MDS therapeutic azacitidine. The effects of DNA methyltransferase inhibition are likely due to a combination of their effects on the microenvironment and on the hematopoietic stem cell compartment. Other MDS therapeutics such as lenalidomide are also known to target the microenvironment [88]. For example, mesenchymal stromal cells treated with lenalidomide secrete less CXCL12, a chemokine involved in hematopoietic stem cell retention within the niche, and lenalidomide can restore the supportive role of low grade MDS-MSCs on the erythroid and myeloid differentiation potential of hematopoietic stem cells [106].

Recent studies point to a possible common clonal origin for MSCs and myeloid neoplasia [129], in this case in the setting of systemic mastocytosis. The characteristic *KIT D816V* mutation was found in the mesenchymal stem cell compartment in a quarter of cases, and those patients had a more aggressive disease course. It is possible then that expansion of a clonally related mesenchymal stem cell compartment contributes to disease progression in myeloid neoplasms. Systemic mastocytosis is not infrequently associated with other myeloid neoplasms, including MDS; whether there is shared clonal origin among the systemic mastocytosis, the non-mast cell myeloid neoplasm, and the mesenchymal stem cell compartment remains to be elucidated. Transfer of coding genetic information via microvesicles, such as transfer of microRNAs from MSCs to CD34+ HPSCs, may also play a role in the pathogenesis of MDS [130].

Molecular interrogation of the microenvironmental compartment is of great research interest, but for practical reasons is unlikely to become relevant to clinical practice in the near future. Tools for assessing the extent and topological interrelationships of various microenvironmental compartments however are readily available through microscopy and readily implementable in clinical practice, and knowledge of topobiology can moreover serve to guide and make sense of *in vitro* and mouse model research.

We have previously demonstrated expansion of the CD271+ mesenchymal stromal cell compartment in poor risk MDS [96]; immunohistochemical analysis of this stromal compartment is readily performed on routine paraffin-embedded bone marrow core biopsies, and can be performed on archival tissue. Reticulin and trichrome stains to assess marrow fibrosis are already a routine component of diagnostic bone marrow evaluation; MDS patients with severe fibrosis have worse bone marrow failure-related death, increased risk of AML transformation, and worse outcomes post transplant [131]. Symptomatic anemia results in transfusion dependence in some 40% of MDS patients, leading to significant increases in morbidity and attendant costs [132]. Iron chelation therapy improves erythroid, platelet and neutrophil cytopenia in transfusion-dependent patients with MDS [133], perhaps by ameliorating reactive oxygen species-related damage to both hematopoietic stem cells and the mesenchymal stem cell compartment [134]. We have shown that marrow iron stores are increased in MDS marrow independent of transfusion history, and indeed correlates with shorter survival independent of IPSSR and transfusion history [62]. Marrow iron is assessed routinely as part of the diagnostic bone marrow evaluation for MDS, and its quantification can confer additional prognostic information at no additional cost.

## 7. Conclusions

In hematopoiesis, “niche” and “microenvironment” are both terms that describe the interactions among hematopoietic elements and their surrounding environment. It is unclear whether these are interchangeable or complementary terms. The microenvironment concept, as elegantly presented by Dr. Pierre Charbord [135], originates from the pioneer experiments of Wolf and Trentin in the 1970s [136], which challenged the stochastic model proposed by Till *et al.* (1964) [137]. The authors found that HPSCs followed a non-random differentiation pathway determined by external factors. They found that the spleen supported more erythroblastic-predominant hematopoiesis than did the bone marrow [138]. La Pushin and Trentin identified differences in the composition of the microenvironment at the two sites, showing that the spleen had a higher content of macrophages than the bone marrow, which had more “stellate” cells (most probably MSCs) [138]. The microenvironment concept was then proven *in vitro*, thanks to Michael Dexter (1977) [139], who established an *in vitro* system for culturing hematopoietic cells. Dexter found that in these cultures, an adherent cell layer (stromal cells) formed after the first weeks of culture, which produced regulatory molecules that were able to support hematopoiesis for the long term (long term marrow cultures Dexter type). Neither of these concepts took into consideration the structures of the bone marrow or the location of the hematopoietic elements within.

**Figure 5:**
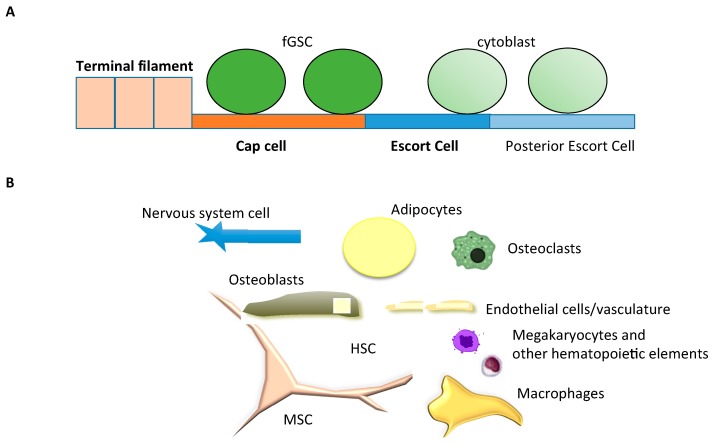
Comparison of niche components in the female germinal stem cell (fGSC) niche in Drosophila and the hematopoietic stem cell (HSC) niche in mouse: (**A**) The fGSC is a fixed niche, composed of three niche cells (terminal filament, cap cells and escort cells). Only cap cells contact fGSC directly; the rest interact with fGSC indirectly. Only fGSC and not differentiated cytoblasts contact cap cells; (**B**) The HSC is a complex niche composed of at least 9 different cell types that impact HSC biology. Mesenchymal stromal cells (MSC) are the cells with the most extensive and pervasive contact, but other cell types also contact HSCs.

The term “topobiology” was first coined by the Nobel Prize awardee Gerald Edelman in his book published in 1988 [140], and his Scientific American publication in 1989 [1]. Herein we apply the concept of topobiology to the niche, the region of the bone marrow where HSCs reside and are regulated. The microarchitecture of the bone marrow is not fixed like the canonical Drosophila fGSC niche, but resembles more closely the concept of the Grinellian or ecological niche [17]. HSCs inhabit varying ecologic zones (e.g., trabecular and long bones, varying marrow composition with age), and they “cohabit” with different hematopoietic cells (lineages and hierarchy). It is difficult in this context to fit a model in which a stem cell contacts a restricted and required set of specific niche cells to avoid differentiation signals, as in the fGSC niche in Drosophila (Figure 5A). In contrast, HSCs contact a variety of stromal and hematopoietic cells, all of which have been demonstrated to impact their physiology (Figure 5B). As the bone marrow is dynamic, the output differs according to physiologic need; the niche and its microenvironment do not have a fixed composition but rather react according to the body’s needs.

**Figure 6:**
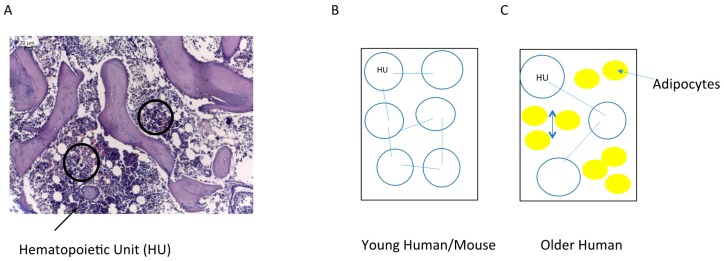
Hematopoietic Unit (HU) model. (**A**) The topobiology of the bone marrow in both mice and humans is composed of several HUs; (**B**) In young humans and mice, the HUs are abundant and have physical contact in the context of highly cellular bone marrow; (**C**) In older humans, the HUs are less abundant and may be separated by adipocytes. Each HU is highly vascularized, and HUs may interact with each other via the vasculature and/or perivascular mesenchymal stromal/stem cells (MSCs).

**Figure 7:**
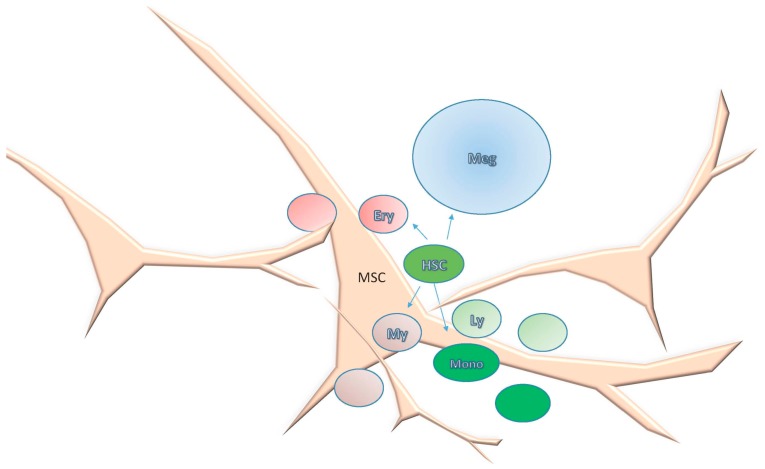
Model of hematopoietic differentiation within a Hematopoietic Unit (HU). Mesenchymal stromal cells (MSCs) are key components of HU; they contact hematopoietic stem cells (HSC) and their progeny as well as the other components of the stem cell niche. Differentiation occurs according to the topobiology of the progenitors, Erythroid (Ery), Megakaryocytic (Meg), Myeloid (My), Lymphoid (Ly) and Monocytic (Mono). MSCs may act as scaffolds that help progenitors to localize in particular regions and to direct their contact with other microenvironmental cells.

This dynamic equilibrium concept is compatible with a model where each bone contains a specific number of repeating hematopoietic stem cell-containing units, a fractal structure as proposed by Naeim F [141] but one whose constituents vary with age, repair or disease (Figure 6). The topobiology model considers that cells respond to their surroundings and that stromal and hematopoietic cells are not in a fixed position. MSCs are a key component of a hematopoietic unit (HU), within which they are positioned to act like sensors and integrators of multiple contact and chemokine-driven signals as they contact HSCs but also lymphoid, myeloid and megakaryocytic cell lineages, adipocytes, macrophages, and blood vessels. They can be compared to neuronal prolongations sensing for inflammatory signals, growth factors, and other cellular elements and integrating those signals (Figure 7). HPSCs are regulated according to their topobiology, which is dynamic and interactive. Each HU is highly vascularized; the vasculature not only delivers nutrients and oxygen but also functions as communication channels among HUs.

**Figure 8:**
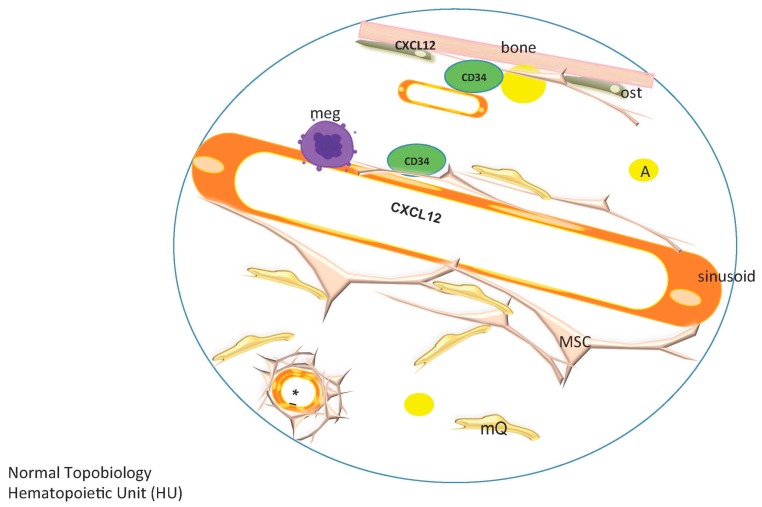
Topobiology of a normal Hematopoietic Unit (HU) in humans. In a normal HU, mesenchymal stromal/stem cells (MSCs) are located in paratrabecular and perivascular areas, and to a lesser extent in the parenchyma. Osteoblast number decreases with age. Megakaryocytes (meg) are located adjacent to sinusoids, MSCs form an outside layer on the sinusoids. Arterioles and capillaries are covered sequentially by pericytes/vascular smooth muscle and a full and thick layer of MSCs, and are often rimmed by plasma cells. Macrophages (mQs) are as abundant as MSCs, and are scattered throughout the bone marrow in perivascular, paratrabecular and parenchymal areas. CD34+ hematopoietic progenitor/stem cells (HPSCs) are located in perivascular areas, with fewer at paratrabecular regions. CXCL12 is produced predominantly by endothelial cells and by osteoblasts.

In this model a hematopoietic subunit includes an HSC and all of its progeny (myeloid, lymphoid, megakaryocytic and erythroid lineages), vessels, MSCs, macrophages, endothelial cells and osteoblasts and osteoclasts (according to the species, strain, and age of the organism) (Figure 8).

**Figure 9:**
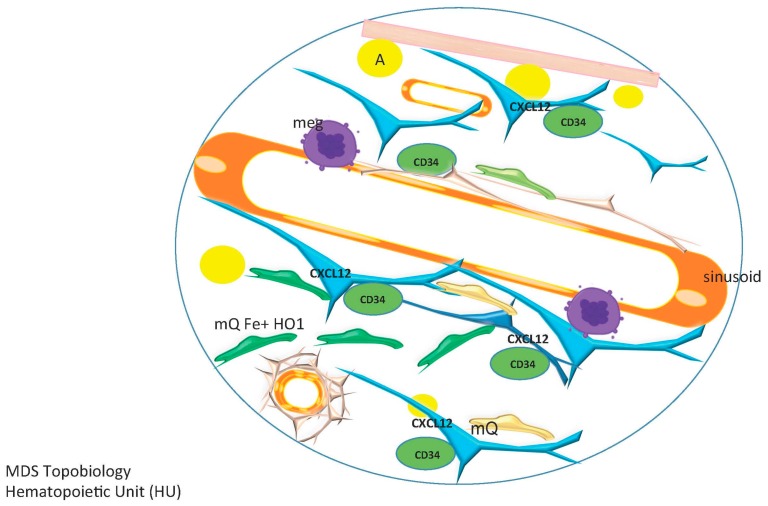
Topobiology of a Myelodysplastic Syndrome (MDS) Hematopoietic Unit (HU). In MDS patients there is a decrease in osteoblasts (accompanied by decreased CXCL12 in paratrabecular areas) and an increase and redistribution of MSC density such that the parenchyma is traversed by a dense MSC meshwork that aberrantly expresses CXCL12. The change in the distribution and secretion pattern of MSCs leads to a re-location of CD34+ hematopoietic progenitor stem/ cells (HPSCs) to the parenchyma, where they are in the proximity of increased iron overloaded macrophages (mQs) overexpressing heme oxygenase-1 (HO1).

The number of HUs and perhaps their composition may vary according to age and disease; a child’s bone marrow is highly cellular, so the spacing of the HUs would be expected to be relatively dense. As the organism ages, the number of HUs diminishes and they become separated by adipocytes (Figure 6B,C). In MDS, there is an increase in the density of subunits; their contact to one another and their composition will lead to aberrant hematopoiesis. Two of the main components of the hematopoietic units, MSCs and macrophages, are increased in density and produce altered cytokines; iron load is also increased, possibly increasing oxidative stress within the hematopoietic unit. B cell precursors, or hematogones, are decreased in MDS [70] and thus are likely depleted within the dysfunctional MDS hematopoietic subunit (Figure 9).

## Figures and Tables

**Table 1:** Summary of *in vitro*, murine *in vivo*, and human *in situ* data on MDS microenvironment.

Factor	Stromal Cultures	MSC	Macrophages
**TNFα**	increased	normal	increased
IL-1B	increased	increased	–
IL-6	increased	normal	–
IFNγ	increased	increased	increased-subset
TGFβ	increased	increased (HR)	correlate with number
SCF	–	increased (2) reduced (2)	–
CXCL12/SDF1	–	*increased (2)* reduced (3)	–
Hematopoietic support	normal (1) deficient (1)	normal (2) deficient (6)	–
Proliferation	normal	normal (5) deficient (7)	NA
CFU-F	NA	normal (1) deficient (3)	NA
Karyotype	–	abnormal	–
Osteoblasts	NA	normal (5) *abnormal (9)*	NA
Adipocytes	NA	normal (4) abnormal (3) higher (1)	NA
Immunomodulation	NA	normal (1) abnormal (3)	NA

Number of supportive publications in parentheses; *in situ* evidence in bold. CFU-F, colony-forming unit fibroblast; CXCL, C-X-C motif chemokine ligand; IL, interleukin; (NA) not applicable; SDF, stromal cell-derived factor; TGF, transforming growth factor; TNF, tumor necrosis factor*. In situ* evidence in italics.
